# A brief comparison of human factor XII-Ala^188^ and factor XII-Pro^188^

**DOI:** 10.1016/j.rpth.2025.102957

**Published:** 2025-07-16

**Authors:** Aleksandr Shamanaev, David Gailani

**Affiliations:** Department of Pathology, Microbiology and Immunology, Vanderbilt University Medical Center, Nashville, Tennessee, USA

Factor (F)XII is the zymogen of the plasma protease FXIIa [[1], [2], [3]]. As part of the plasma kallikrein–kinin system (KKS), FXII undergoes reciprocal activation with the zymogen prekallikrein (PK), generating the proteases FXIIa and plasma kallikrein (PKa), respectively [1,4,5]. PKa then cleaves high-molecular-weight kininogen (HK) to release the vasoactive peptide bradykinin [6,7]. KKS dysregulation causes excessive bradykinin generation in most forms of the inherited disorder hereditary angioedema (HAE) [[8], [9], [10]]. Acceleration of reciprocal activation also occurs when blood comes into contact with a variety of biological and nonbiological surfaces, such as during cardiopulmonary bypass, renal dialysis, and extracorporeal membrane oxygenation [11,12].

The gene for human FXII (*F12*) is located on the long arm of chromosome 5. The cDNA for human FXII described by Cool et al. [3] in 1985 encodes an 80-kDa multidomain glycoprotein with a trypsin-like catalytic domain at its C-terminus. The mature plasma protein derived from this cDNA has a proline residue at amino acid position 188 within the second epidermal-growth factor domain. For this discussion, we will refer to this protein as FXII-Pro^188^. Factor XII-Pro^188^ was assumed to be the predominant wild-type form of FXII for many years, and several structure–function analyses have been conducted with proteins expressed from the cDNA described by Cool et al. [4,13,14]. It has subsequently been determined that *F12* genes encoding FXII-Pro^188^ represent a minor allele, with the most common *F12* allele in humans encoding alanine at position 188 (FXII-Ala^188^) [15].

There is no published evidence demonstrating that FXII-Ala^188^ and FXII-Pro^188^ differ functionally. However, Pechnikova et al. [16] recently described a 13-year-old girl with symptoms of HAE who is homozygous for the allele encoding FXII-Pro^188^ and speculated that the Pro^188^ form of FXII may contribute to her symptoms [16]. The patient’s father, who does not exhibit symptoms of HAE, is also homozygous for FXII-Pro^188^, while her mother is heterozygous for this polymorphism. While most cases of HAE are associated with reduced plasma levels of the serpin C1-inhibtor (C1-INH) [6,8,17], amino acid substitutions in FXII have been linked to HAE and inflammatory syndromes [9]. A Thr^309^ to Lys or Arg replacement in the FXII proline-rich region has been identified in some patients with HAE with normal C1-INH levels [4,9,18,19]. The substitutions introduce a novel protease cleavage site that facilitates conversion of full-length FXII to a truncated form (ΔFXII) that is activated >10 times faster than the full-length protein [4,9,19]. A Trp^268^ to Arg substitution in the FXII kringle domain causes an inherited cold-induced urticarial autoinflammatory syndrome [20,21]. Both mutations result in FXII assuming a conformation that renders it more susceptible to activation by PKa [4,9]. The recent case report raised the possibility that FXII-Pro^188^ is another example of such an open form of FXII.

cDNAs encoding FXII-Ala^188^ and FXII-Pro^188^ differ only in a G-to-C substitution in the first base pair of the GCC triplet encoding Ala^188^, resulting in CCC encoding Pro^188^. A comparison of FXII amino acid sequences from vertebrate species reveals heterogeneity at this location [22]. Arginine is present in most mammals including the chimpanzee, while proline is found in the baboon, a reptile (painted turtle) and an amphibian (high Himalayan frog) (Figure 1A). The *F12* gene is the result of a duplication of the hepatocyte growth factor activator gene (*HGFA*), and arginine is also present at the position in hepatocyte growth factor activator equivalent to FXII^188^ (Figure 1A) [23].

**Figure 1:**
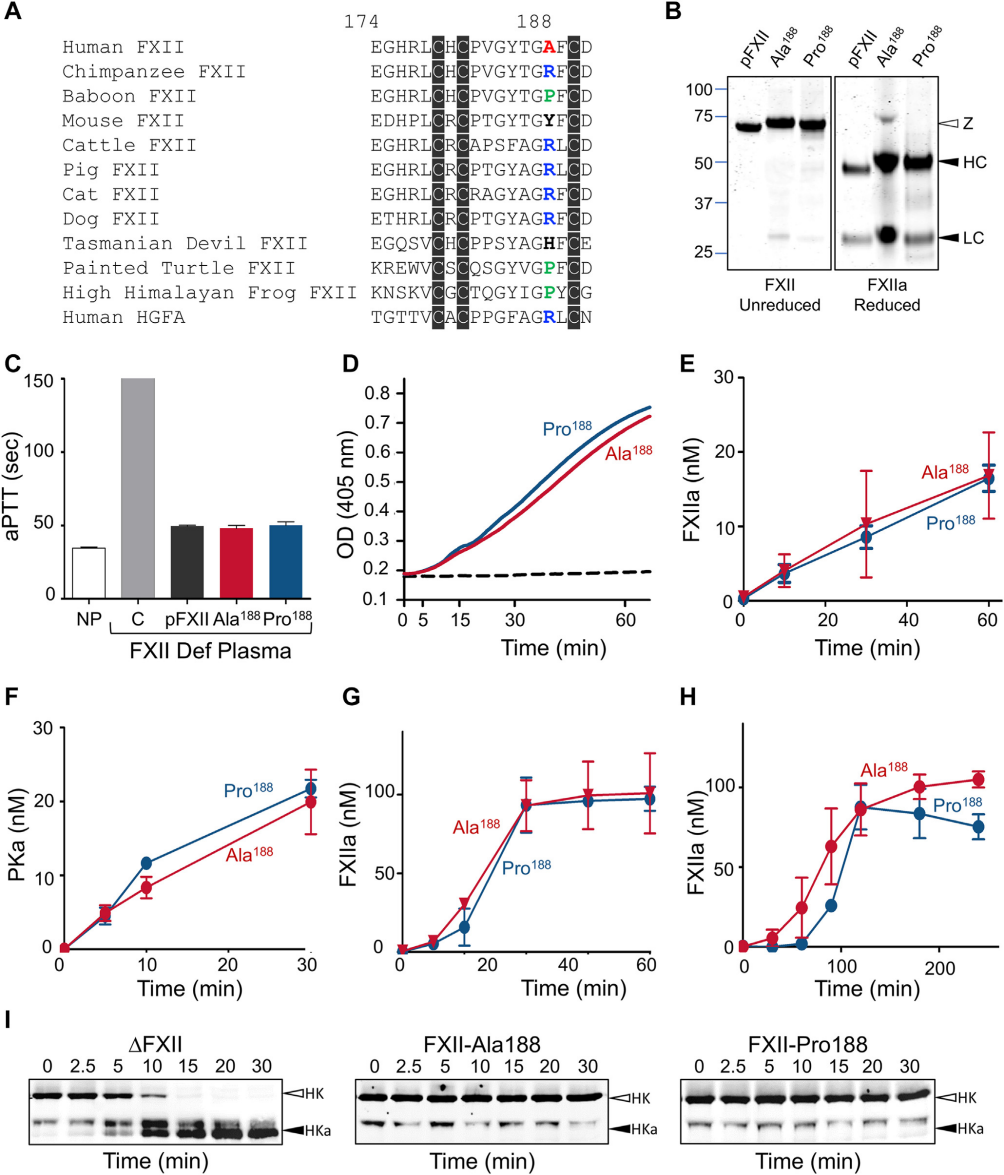
Factor (F)XII Ala/Pro^188^ activation and activity. (A) Amino acid sequences of FXII epidermal-growth factor 2 domain residues 174 to 191 (human FXII numbering system) in vertebrate species showing heterogeneity at residue 188. (B) Coomassie Blue stained polyacrylamide gels of 2 μg samples of zymogen (left panel, nonreduced) or activated (right panel, reduced) plasma-derived FXII (pFXII), and recombinant FXII-Ala^188^ (Ala^188^) and FXII-Pro^188^ (Pro^188^). Positions of molecular mass standards are on the left, and standards for zymogen FXII (Z) and the heavy (HC) and light (LC) chains of FXIIa are shown to the right. (C) Activated partial thromboplastin times (aPTT) of normal human plasma (NP), FXII-deficient human plasma (C), and FXII-deficient plasma supplemented with 400 nM plasma or recombinant FXII. (D) Reciprocal activation of 60 nM prekallikrein (PK) with 12.5 nM FXII-Ala^188^ (red), FXII-Pro^188^ (blue), or vehicle (dashed line) in the presence of 200 μM chromogenic substrate S-2302. Generation of plasma kallikrein (PKa) and FXIIa was followed by monitoring at OD 405 nm on a spectrophotometer. (E) Activation of 100 nM FXII-Ala^188^ (red) or FXII-Pro^188^ (blue) by 12.5 nM PKa. At indicated times, samples were removed, PKa was inhibited with 500 nM soybean trypsin inhibitor, and FXIIa concentration was determined by chromogenic substrate assay. Results were compared with a curve generated with known FXIIa concentrations. (F) PK (60 nM) activation by 120 pM FXIIa-Ala^188^ (red) or FXIIa-Pro^188^ (blue). At indicated times, samples were removed, FXIIa was inhibited with 500 nM corn trypsin inhibitor, and PKa concentration was determined by chromogenic substrate assay. Results were compared with a curve generated with known PKa concentrations. (G, H) FXII autoactivation. FXII-Ala^188^ (red) or FXII-Pro^188^ (blue) (100 nM) were incubated with (G) 1 μg/mL dextran sulfate (500,00 Da) or (H) 70 μM long-chain polyphosphate. At indicated times, samples were removed into polybrene to dissociate FXII/FXIIa from dextran sulfate or plyphosphate, and FXIIa was measured by chromogenic substrate assay. (I) Western blots for high-molecular-weight kininogen (HK) in human plasma. FXII–deficient plasma was supplemented with 140 nM ΔFXII [4,9], FXII-Ala^188^, or FXII-Pro^188^ and incubated at 37 °C. At indicated times, aliquots were removed into nonreducing sample buffer, size fractionated by sodium dodecyl-sulfate polyacrylamide gel electrophoresis, and transferred to nitrocellulose membranes. Blots were developed with a goat antihuman HK IgG. Positions of markers for uncleaved HK (white arrow) and fully cleaved HK (HKa, black arrow) are indicated to the right of each image. HGFA, hepatocyte growth factor activator.

Given the recent case report [16], we felt it is important to determine whether FXII-Pro^188^ has an enhanced ability to activate the KKS compared with FXII-Ala^188^, as reported for FXII-Lys/Arg^309^ [4] and FXII-Arg^268^ [20,21]. Factor XII-Ala^188^ and FXII-Pro^188^ were expressed in HEK293 cells and purified from conditioned media (Figure 1B, left panel) [4,24]. Their zymogen forms reconstitute FXII-deficient plasma similarly in an activated partial thromboplastin time assay (Figure 1C). When mixed in solution, FXII and PK convert each other to the proteases FXIIa and PKa [4,5]. Several FXII variants with single amino acid substitutions have been identified that accelerate this process [1,4,5,20,24]. There was no obvious difference between FXII-Ala^188^ and FXII-Pro^188^ in their capacities to support reciprocal activation (Figure 1D). During reciprocal activation, PKa activates FXII to FXIIa and FXIIa, in turn, converts PK to PKa [1,2,4,5,24]. Factor XII-Ala^188^ and FXII-Pro^188^ were activated by PKa at similar rates (Figure 1E), and FXIIa-Ala^188^ and FXIIa-Pro^188^ (Figure 1B, right panel) activated PK similarly (Figure 1F). Factor XII undergoes autocatalytic activation when bound to anionic polymers such as dextran sulfate and polyphosphate [1,4,11,12]. In the presence of dextran sulfate (Figure 1G) or long-chain polyphosphate (Figure 1H, 70 μM), FXII-Ala^188^ and FXII-Pro^188^ auto-convert to FXIIa similarly. In hereditary angioedema caused by FXII Thr^309^Lys/Arg substitutions, formation of truncated ΔFXII enhances reciprocal activation with PK, leading to rapid HK cleavage and bradykinin production [4,9,19]. Adding ΔFXII to human FXII-deficient plasma leads to rapid HK cleavage (Figure 1I, left panel) [4,9,19,24]. In contrast, adding FXII-Ala^188^ (Figure 1I, middle panel) or FXII-Pro^188^ (Figure 1I, right panel) to FXII-deficient plasma has no perceptible effect on HK.

Our analysis did not identify an obvious gain-of-function phenotype in FXII-Pro^188^ relative to FXII-Ala^188^, which could contribute to HAE by enhancing reciprocal activation with PK. These reassuring results indicate that several recent structure–function analyses that used FXII with a proline at position 188 ^4^^,^^9^^,^^13^^,^^14^^,^[19], [20], [21] are likely to yield similar results if FXII-Ala^188^ is used as the wild-type protein [24]. This does not rule out the possibility that FXII-Pro^188^ is contributing in some unanticipated way to HAE in the recently reported patient [16]. However, it should be noted that both of her parents have this polymorphism (one a homozygote) without exhibiting symptoms of HAE. The minor *F12* allele encoding FXII-Pro^188^ is still relatively common, with an estimated frequency of 1% to 2%. We are not aware of other studies linking FXII-Pro^188^ to HAE, although it may be worth testing for it in analyses of patients with HAE with normal plasma C1-INH levels to see whether it is enriched relative to the general population. Regardless of the impact of Pro^188^ on FXII function, it seems reasonable, going forward, to consider FXII-Ala^188^ to be wild-type FXII for purposes of structure–function and enzymatic analyses as it is the predominant form of FXII in humans.

## References

[1] Shamanaev A., Litvak M., Ivanov I., Srivastava P., Sun M.F., Dickeson S.K. (2024). Factor XII structure-function relationships. Semin Thromb Hemost.

[2] de Maat S., Maas C. (2016). Factor XII: form determines function. J Thromb Haemost.

[3] Cool D.E., Edgell C.J., Louie G.V., Zoller M.J., Brayer G.D., MacGillivray R.T. (1985). Characterization of human blood coagulation factor XII cDNA. Prediction of the primary structure of factor XII and the tertiary structure of β-factor XIIa. J Biol Chem.

[4] Ivanov I., Matafonov A., Sun M.F., Mohammed B.M., Cheng Q., Dickeson S.K. (2019). A mechanism for hereditary angioedema with normal C1 inhibitor: an inhibitory regulatory role for the factor XII heavy chain. Blood.

[5] Shamanaev A., Litvak M., Gailani D. (2022). Recent advances in factor XII structure and function. Curr Opin Hematol.

[6] Schmaier A.H. (2016). The contact activation and kallikrein/kinin systems: pathophysiologic and physiologic activities. J Thromb Haemost.

[7] Maas C., Renné T. (2018). Coagulation factor XII in thrombosis and inflammation. Blood.

[8] Schmaier A.H. (2018). Plasma prekallikrein: its role in hereditary angioedema and health and disease. Front Med (Lausanne).

[9] Shamanaev A., Dickeson S.K., Ivanov I., Litvak M., Sun M.F., Kumar S. (2023). Mechanisms involved in hereditary angioedema with normal C1-inhibitor activity. Front Physiol.

[10] Lacuesta G., Betschel S.D., Tsai E., Kim H. (2024). Angioedema. Allergy Asthma Clin Immunol.

[11] Jaffer I.H., Weitz J.I. (2019). The blood compatibility challenge. Part 1: blood-contacting medical devices: the scope of the problem. Acta Biomater.

[12] Litvak M., Shamanaev A., Zalawadiya S., Matafonov A., Kobrin A., Feener E.P. (2023). Titanium is a potent inducer of contact activation: implications for intravascular devices. J Thromb Haemost.

[13] Heestermans M., Naudin C., Mailer R.K., Konrath S., Klaetschke K., Jämsä A. (2021). Identification of the factor XII contact activation site enables sensitive coagulation diagnostics. Nat Commun.

[14] Shamanaev A., Ivanov I., Sun M.F., Litvak M., Srivastava P., Mohammed B.M. (2022). Model for surface-dependent factor XII activation: the roles of factor XII heavy chain domains. Blood Adv.

[15] Benson D.A., Karsch-Mizrachi I., Lipman D.J., Ostell J., Rapp B.A., Wheeler D.L. (2000). GenBank. Nucleic Acids Res.

[16] Pechnikova N., Yaremenko A.V., Saitgalina M.A., Shchemelev A.N., Bebyakov A.M., Denisova A.R. (Posted online October 4, 2023). In silico analysis and in-depth assessment of a female patient with a missence mutation in the F12 gene associated with hereditary angioedema: a case study. Research Square.

[17] Costanzo G., Sambugaro G., Firinu D. (2024). Hereditary angioedema due to C1-inhibitor deficiency: current therapeutic approaches. Curr Opin Allergy Clin Immunol.

[18] Bork K. (2013). Hereditary angioedema with normal C1 inhibitor. Immunol Allergy Clin North Am.

[19] de Maat S., Clark C.C., Boertien M., Parr N., Sanrattana W., Hofman Z.L.M. (2019). Factor XII truncation accelerates activation in solution. J Thromb Haemost.

[20] Hofman Z.L.M., Clark C.C., Sanrattana W., Nosairi A., Parr N.M.J., Živkovic M. (2020). A mutation in the kringle domain of human factor XII that causes autoinflammation, disturbs zymogen quiescence, and accelerates activation. J Biol Chem.

[21] Scheffel J., Mahnke N.A., Hofman Z.L.M., Maat S., Wu J., Bonnekoh H. (2020). Cold-induced urticarial autoinflammatory syndrome related to factor XII activation. Nat Commun.

[22] Uniprot Consortium (2019). UniProt: a worldwide hub of protein knowledge. Nucleic Acids Res.

[23] Miyazawa K., Shimomura T., Kitamura A., Kondo J., Morimoto Y., Kitamura N. (1993). Molecular cloning and sequence analysis of the cDNA for a human serine protease responsible for activation of hepatocyte growth factor. Structural similarity of the protease precursor to blood coagulation factor XII. J Biol Chem.

[24] Shamanaev A., Ma Y., Ponczek M.B., Sun M.-F., Cheng Q., Dickeson S.K. (2025).

